# On the Medical and Surgical Uses of Electricity

**Published:** 1891-12

**Authors:** 


ON THE MEDICAL AND SURGICAL USES OF ELECTRICITY. By GEO. M. BEARD, A. M., M. D., and A. D. ROCKWELL, A. M., M. D., Formerly Professor of Electro-Therapeutics in the New York Post-Graduate Medical School and Hospital; Fellow of the New York Academy of Medicine; Member of the American Academy of Medicine; Member of the New York Neurological Society; formerly Electro-Therapeutist to the Woman's Hospital of the State of New York, etc. Eighth edition, with over two hundred illustrations. 788 pp., cloth. New York: William Wood & Co.

This work has been before the public for over ten years, and in that time it has established a place for itself in electro-therapeutic literature.

The volume is divided into four parts, treating respectively of electro-physics, electro-physiology, electro-therapeutics, and electro-surgery. In the section devoted to electro-physics, magnets, frictional, galvanic and faradic electricity, are discussed, and the properties of each, and method of producing them, explained. Ohm's law, and its practical relation to electro-therapeutics, has a well-written chapter devoted to it.

In the section on electro-physiology, the action of electricity on the skin, brain, spinal cord, sympathetic, pneumogastric optic, auditory and gustatory nerves, is detailed with great care, and supported by the experiments of such men as Brenner, Fenier, Hitzig, Claude Bernard, and others.

Other chapters in this section treat of the effect of electricity on voluntary and involuntary muscles on the motor and sensory nerves. This section is very complete, and of great importance, as no one can properly use a therapeutic agent until he understands its physiological action, and surely electricity is no exception to this rule.

When we take up the section on electro-therapeutics, we are impressed with the number of diseases for which electricity has been employed, and the variety of effects which may be expected from the various methods of application.

A strong plea is made for the tonic property of central galvanization and general faradization.

We quote from page 200: "The very marked permanent effect is improvement in sleep. They permanently improve the appetite and digestive capacity, and regulate the bowels." Agreeing, as we do, with the authors, that these effects result from electrical treatment, we must dissent from the statement that the improvement is permanent.

When we come to the treatment of special diseases, we must admit that in some instances we are puzzled as to the authors' meaning, as, on page 578, we read of infantile paralysis: "There is probably no one pathological lesion that is pathognomonic of the disease." We have been of the opinion that it was now an accepted fact that the lesion in the anterior cornea was a pathognomonic one.

In many instances the authors will refer to the use of electricity for a certain condition, and then report a single case, as, on page 613, we read that "Ptosis is to be treated like spasm of the lid (its opposite condition), but with a stronger current." A single case is then reported of ptosis, following herpes, cured under galvanization. This was a case of ptosis due to an acute affection, and belonged to the class of readily curable cases. But the reader should not be left to infer that, as a rule, ptosis is easily cured, for it is not, the third nerve being very difficult to treat.

On reading the page devoted to Paralysis Agitans, we read that there are two kinds: 1st. Those with organic lesions. 2d. Those where no functional lesions can be discovered. Of the treatment of this progressively incurable affection, we read, "that central galvanization and general faradization may be used with benefit. The best results have been obtained by galvanization of the spine and sympathetic and brain." "The best results" is a misleading expression, as, unfortunately, no results in paralysis agitans are entitled to the words, best results.

If there is any one criticism which we think is especially called for, it is the general looseness of expression when speaking of results. In the page given to epilepsy, three cases are reported, one marked apparent recovery. Of this patient we read that when they received the patient, she had been taking the bromides for several years, and they then gave her Brown-Sequard's formula, and electrical treatment; result, apparent recovery. This is not a fair case for us to quote as a proof that electricity will benefit epilepsy.

In the last section, devoted to electro-surgery, much of interest and value is found. The technique of electrolysis for the various growths, and the use of the galvano-cautery, are admirably presented.

The book, as a whole, is one of the most complete of its kind, and one can hardly fail to find within its pages anything on medical electricity, in all its branches.

Most of us will not agree with the authors in all their fond hopes, and for that reason we shall be less liable to disappointment.

The illustrations and type are excellent. J. W. P.

